# Crystal structure of trimeth­yl({tris­[(phenyl­sulfan­yl)meth­yl]sil­yl}meth­oxy)silane and Hirshfeld surface analysis of 3-bromo-2,2-bis­(bromo­meth­yl)propan-1-ol

**DOI:** 10.1107/S205698902300227X

**Published:** 2023-03-15

**Authors:** Jan Frederick Wappelhorst, Jonathan Wattenberg, Carsten Strohmann

**Affiliations:** a Technische Universität Dortmund, Fakultät Chemie und Chemische Biologie, Otto-Hahn-Strasse 6, 44227 Dortmund, Germany; Katholieke Universiteit Leuven, Belgium

**Keywords:** crystal structure, thio­ether ligand, Hirshfeld surface analysis

## Abstract

Trimeth­yl({tris­[(phenyl­sulfan­yl)meth­yl]sil­yl}meth­oxy)silane (**3**), C_26_H_32_OS_3_Si, is a new ligand for transition-metal coordination chemistry derived from 3-bromo-2,2-bis­(bromo­meth­yl)propan-1-ol (**1**), C_5_H_9_Br_3_O, through silylation and following exchange of bromine groups with NaSPh. Silylated thio­ether ligand **3** crystallizes in the centrosymmetric space group *C*2/*c*. Bromo­methyl­alcohol **1** crystallizes in the space group *P*  in the triclinic crystal system with four mol­ecules in the asymmetric unit. Analysis of the Hirshfeld surface shows structure-defining inter­actions for bromo­methyl­alcohol **1**, resulting in inter­molecular hydrogen bonds between the hydroxyl groups along the *a*-axis direction.

## Abstract 2

Trimeth­yl({tris­[(phenyl­sulfan­yl)meth­yl]sil­yl}meth­oxy)silane (**3**) is a new ligand for transition-metal coordination chemistry derived from 3-bromo-2,2-bis­(bromo­meth­yl)propan-1-ol (**1**) through silylation and following exchange of bromine groups with NaSPh. Analysis of the Hirshfeld surface shows structure-defining inter­actions for bromo­methyl­alcohol **1**, resulting in inter­molecular hydrogen bonds between the hydroxyl groups along the *a*-axis direction.

## 1. Chemical context

Thio­ether ligands offer an attractive alternative to known phosphine or amine ligands. As a result of the soft property of thio­ethers, they coordinate to transition metals (Awaleh *et al.*, 2008 ▸; Knaust & Keller, 2003 ▸; Knorr *et al.*, 2012 ▸, 2014 ▸; Schlachter *et al.*, 2022 ▸). Furthermore, because of its two lone pairs, sulfur can act as a bridging ligand between two metal centres and thus favour coordination polymers (Awaleh *et al.*, 2010 ▸; Peindy *et al.*, 2009 ▸; Schlachter *et al.*, 2018 ▸, 2020 ▸, 2021 ▸; Viau *et al.*, 2022 ▸).

In addition, thio­ether ligands are increasingly gaining inter­est for redox catalysis, as their stabilizing effect towards the metal centres differ from those of the common phosphine or amine ligands, and thus new catalytic accesses can be created (Petuker *et al.*, 2017 ▸). 
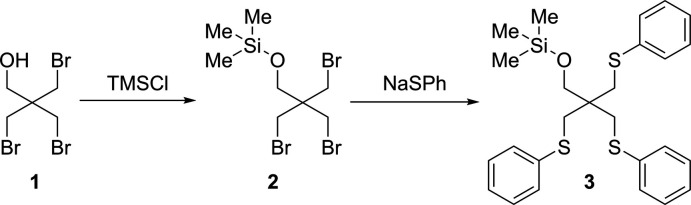


Furthermore, the solubility of ligands in polar and non-polar solvents plays a major role. Polar hydroxyl groups, such as bromo­methyl­alcohol **1**, will reduce solubility in non-polar solvents and can cause problems like the reduced formation of catalytic species in the process. To prevent this, the hydroxyl group was silylated *via* conditions known from the literature, thus increasing the lipophilicity of ligand **3**.

In the following, the structure of bromo­methyl­alcohol **1** and silylated thio­ether ligand **3**, as well as the surface inter­actions of **1** are discussed in terms of Hirshfeld surface analysis.

## 2. Structural commentary

**Figure 1:**
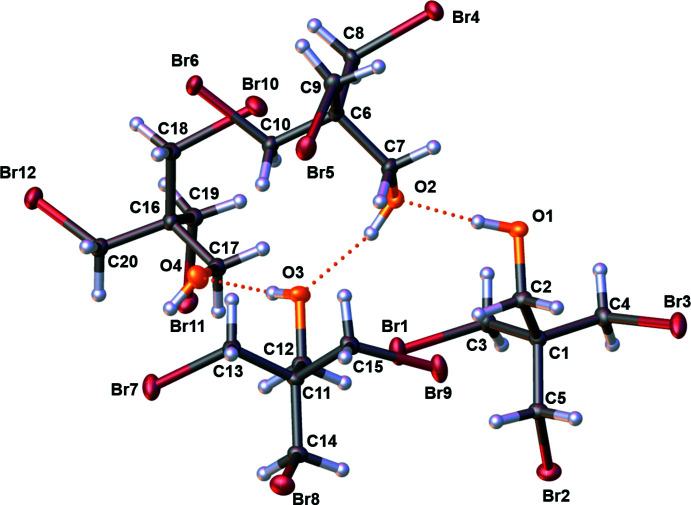
The mol­ecular structure of bromo­methyl­alcohol **1** with displacement ellipsoids drawn at the 50% probability level.

Bromo­methyl­alcohol **1** crystallizes at 243.15 K from diethyl ether in the centrosymmetric space group *P* 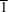 with four mol­ecules present in the asymmetric unit (*Z*′ = 4, *Z* = 2). The mol­ecular structure of bromo­methyl­alcohol **1** is shown in Fig. 1 ▸ and selected bond lengths and angles are given in Table 1 ▸.

The bond lengths to be expected for a C(alk­yl)–Br bond are in the range 1.880–1.940 Å (Allen *et al.*, 1987 ▸). The C(alk­yl)—Br bonds listed in Table 1 ▸ are located in the upper range of these bond lengths. The O1—C2 bond length of 1.432 (3) Å corresponds to an expected length for a C(alk­yl)—OH bond of between 1.393–1.456 Å. Furthermore, the bond angles C1—C3—Br1, C1—C4—Br2 and C1—C5—Br3 are similar and in general comparable in size with similar structural motifs (Bukowska-Strzyżewska & Skoweranda, 1980 ▸).

**Figure 2:**
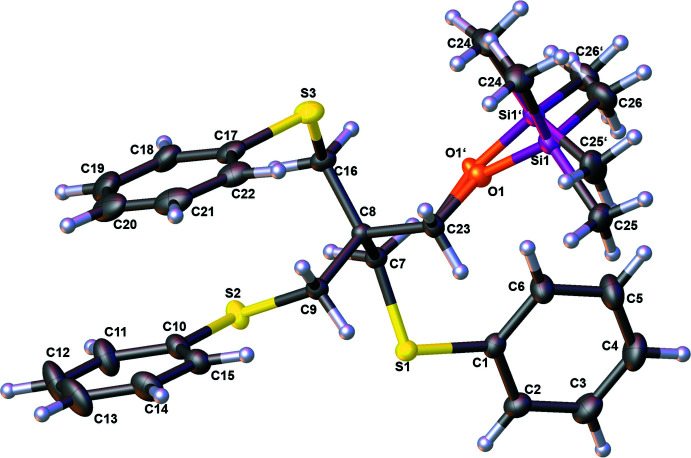
The mol­ecular structure of silylated thio­ether ligand **3** with displacement ellipsoids drawn at the 50% probability level.

The mol­ecular structure of silylated thio­ether ligand **3** is shown in Fig. 2 ▸ and selected bond lengths and angles are given in Table 2 ▸.

The S—C(alk­yl) bonds are of comparable lengths to each other and correspond to the expected bond lengths for alk­yl–sulfur bonds (1.778–1.856 Å; Allen *et al.*, 1987 ▸). The S—C(Ph) bonds, on the other hand, are significantly shorter than the S—C(alk­yl) bonds, but are within the range of expected bond lengths (1.700–1.802 Å). Comparing the bond angles at sulfur to similar structural motifs, the angles are quite similar (Tinant *et al.*, 1987 ▸; Crundwell *et al.*, 1999 ▸). The length of the C—O bond of the ether from silylated thio­ether ligand **3** lies in a comparable range to the -C–O bond from bromo­methyl­alcohol **1**. The occupancies at the disordered TMSO group are 50.9 (3)% for O1/Si1/C24–C26 and 49.1 (3)% for O1′/Si1′/C24′–C26′. The disorder at the TMSO group also shows a shorter O1—C23 [1.384 (15) Å] bond length than O1′—C23 [1.479 (14) Å]. The expected bond length for a **C—O**—Si bond is between 1.365 and 1.467 Å (Allen *et al.*, 1987 ▸). Furthermore, the bond lengths O1—Si1 [1.632 (12) Å] and O1′—Si1′ [1.672 (13) Å] are similar, as are the bond angles between C23—O1—Si1 [123.6°(7)] and C23—O1′—Si1′ [123.7°(7)].

## 3. Supra­molecular features

**Figure 3:**
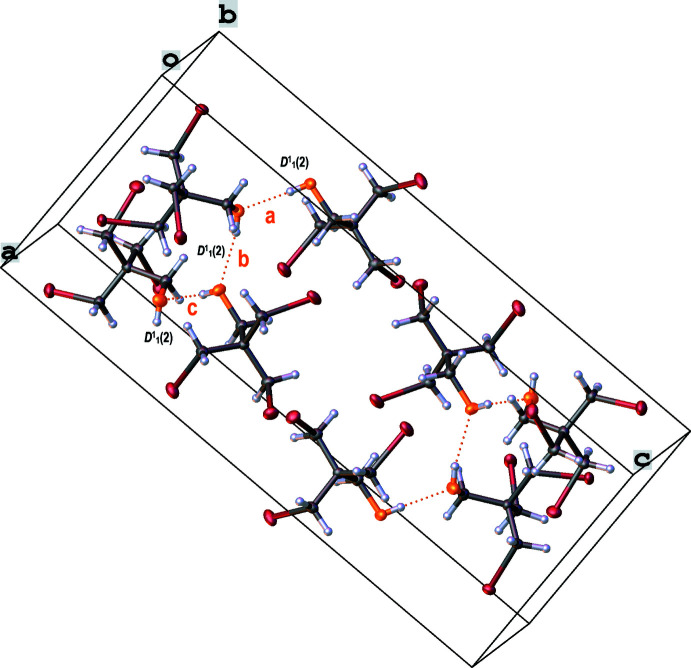
Crystal packing of bromo­methyl­alcohol **1** Hydrogen bonds are shown as dashed lines. Hydrogen bonds a, b and c have the graph-set motif *D* ^1^_1_(2).

The crystal packing of bromo­methyl­alcohol **1** is shown in Fig. 3 ▸ and is defined by inter­molecular hydrogen bonds along the *a*-axis direction, which are given in Table 3 ▸. Here, the contacts between O1—H1⋯O2 [2.712 (3) Å] and O3—H3⋯O4 [2.729 (3) Å] are slightly shorter than those between O2—H2⋯O3 [2.766 (3) Å] and O4—H4⋯O1^i^ [2.773 (3) Å]. Moreover, the angles are approximately linear at 175 (4)° (O3—H3⋯O4) and 168 (4)° (O1—H1⋯O2). These hydrogen bonds can be assigned the graph-set symbol *D* ^1^_1_(2). This means that a hydrogen bond between two adjacent hydroxyl groups, O1—H1⋯O2, is established. The contact between O4—H4⋯O1 is created *via* the symmetry operation (i) *x* − 1, *y*, *z*.

**Figure 4:**
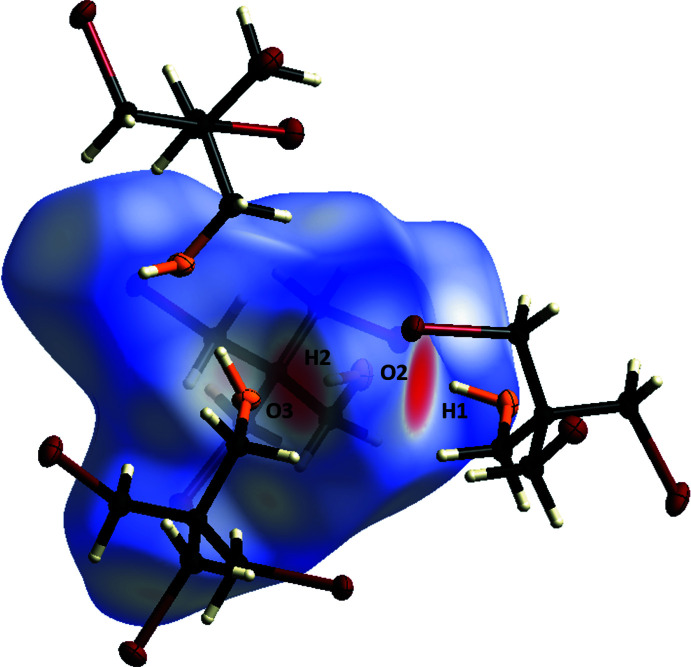
Hirshfeld surface analysis of **1** showing close contacts in the crystal. The hydrogen bonds between H1⋯O2 and H2⋯O3 are labelled.

**Figure 5:**
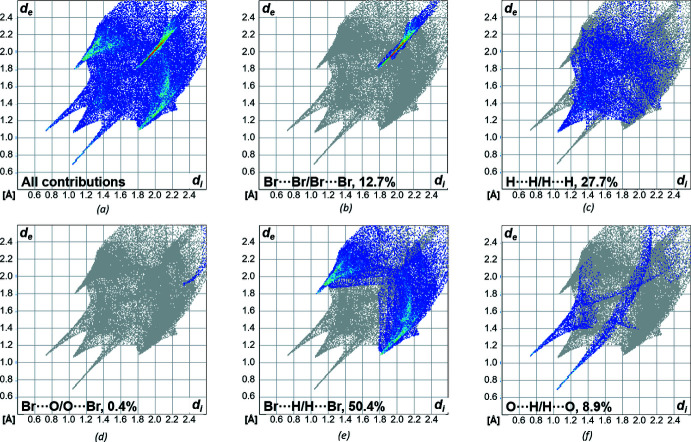
Two-dimensional fingerprint plots for bromo­methyl­alcohol **1** showing close contacts for (*a*) all contributions in the crystal and those delineated into (*b*) Br⋯Br, (*c*) H⋯H, (*d*) Br⋯O/O⋯Br, (*e*) Br⋯H/H⋯Br and (*f*) O⋯H/H⋯O-inter­actions.

To gain further insight into the supra­molecular packing inter­actions, a Hirshfeld surface analysis was performed (Spackman & Jayatilaka, 2009 ▸). The Hirshfeld surfaces and fingerprint plots were generated and analysed using the program *CrystalExplorer21* (Spackman *et al.*, 2021 ▸). The Hirshfeld surface was mapped over *d*_norm_ in the range −0.66 to 1.14 a.u. (Fig. 4 ▸). The contributions of the different inter­molecular inter­actions for **1** are shown in the two-dimensional fingerprint plots (Fig. 5 ▸; McKinnon *et al.*, 2007 ▸). The different strength of the inter­actions is reflected here by the colouring of the surface. The red dots represent close contacts, whereas blue areas represent no contact. The fingerprint plots show that the O⋯H/H⋯O inter­actions account for only 8.9% of the total surface area, although they are probably the strongest contributors to the inter­molecular inter­actions. The largest contribution to the surface inter­actions comes from the Br⋯H/H⋯Br contacts at 50.4%. This is followed by the contributions of the H⋯H/H⋯H contacts (27.7%). There is no contribution to the surface inter­actions by C⋯H/H⋯C contacts, which is mainly due to the fact that the carbon atoms of the CH_2_ groups in question are shielded from the outside by the terminal Br and OH groups so that they cannot make any contribution. The smallest contribution to the surface inter­actions is made by the Br⋯O/O⋯Br contacts (0.4%), which is due to the spatial arrangement of the bromine substituents in relation to the hydroxyl group.

**Figure 6:**
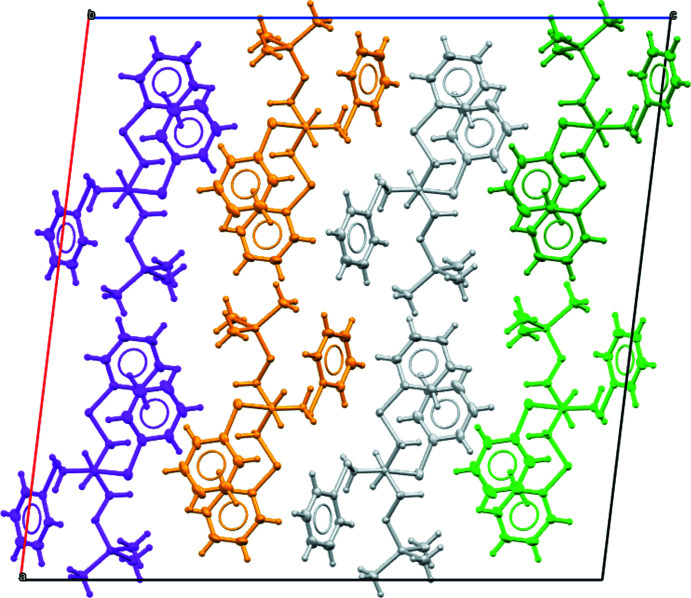
Crystal packing of silylated thio­ether ligand **3** shown along the *b*-axis. Mol­ecules are coloured by their symmetry relationship to the asymmetric unit. The relationships between colour and symmetry are as follows: grey – identity; light green – twofold rotation axis; dark green – twofold screw axis; golden yellow – inversion centre; magenta – glide plane.

The crystal packing of silylated thio­ether ligand **3** is shown in Fig. 6 ▸ and is characterized by propagation along the *b*-axis direction. For silylated thio­ether ligand **3**, apart from the packing effects, there are no overriding inter­molecular inter­actions between the mol­ecules that influences the arrangement of the mol­ecules.

## 4. Database survey

A search of the Cambridge crystallographic database (Groom *et al.*, 2016 ▸; WebCSD January 2023) for 1,3-di­bromo-2-(bro­mo­meth­yl)propanes revealed the following mol­ecules structurally related to bromo­methyl­alcohol **1**: 4-[3-bromo-2,2-bis(bromo­meth­yl)prop­oxy]benzene-1,2-dicarbo­nitrile (VUJ­BOU; Canımkurbey *et al.*, 2020 ▸), 3-[3-bromo-2,2-bis­(bromo­meth­yl)prop­oxy]benzene-1,2-dicarbo­nitrile (VUJBUA; Canımkurbey *et al.*, 2020 ▸) and dimethyl 5-[3-bromo-2,2-bis(bromo­meth­yl)prop­oxy]benzene-1,3-di­carboxyl­ate (BAN­YOI; Najafi Khosroshahi *et al.*, 2021 ▸). For the C—Br bond lengths, similar values are found as in bromo­methyl­alcohol **1**. Furthermore, the C—CH_2_—Br angles observed in **1** corres­pond to the angles in the selected structures.

A search for 1,3-bis­(phenyl­thio)-2-[(phenyl­thio)­meth­yl]propan-2-ol yielded no hits in the WebCSD. By replacing the quaternary carbon with a silicon atom, the comparable structural motif of *catena*-[[μ_2_-tetra­kis­(methyl­thio­meth­yl)silane]di­bromo­mercury(II)] (WAMYUF; Peindy *et al.*, 2005 ▸) was obtained. Here, the C(alk­yl)—S bonds are shorter than in silylated thio­ether ligand **3**. The same applies to the related structure of di­bromo­[tetra­kis­(phenyl­thio­meth­yl)silane-*S*,*S*′]mercury(II) (WAMZAM; Peindy *et al.*, 2005 ▸).

Another related structure motif could be found where the thio­ether groups act as bridging ligands between Cu_2_I_2_ rhomboids (Schlachter *et al.*, 2022 ▸).

## 5. Synthesis and crystallization

Bromo­methyl­alcohol **1** is commercially available and was crystallized at 243.15 K from diethyl ether as clear and colourless plates.

Methyl­lithium (1.6 *M* in *n*-hexane, 16.93 mmol) was dropped into diethyl ether (50 mL) at 273.15 K to **1** (15.39 mmol). The solution was stirred for 1 h at room temperature and then chloro­tri­methyl­silane (16.93 mmol) was added at 273.15 K. It was stirred again for 1 h at room temperature, then the reaction solution was quenched with water. The aqueous phase was extracted three times with di­chloro­methane and the combined organic layers were dried over magnesium sulfate. The volatiles were removed to give compound **2** crude.

^1^H NMR (600 MHz, C_6_D_6_) δ = 3.33 (*s*, 2H; OC*H*_2_C), 3.18 (*s*, 6H; CC*H*_2_Br), 0.03 (*s*, 9H; Si(C*H*_3_)_3_) ppm.

{^1^H}^13^C NMR (151 MHz, C_6_D_6_) δ = 61.6 (1C; O*C*H_2_C), 44.3 [1C; (*C*(CH_2_)_4_], 34.6 (3C; C*C*H_2_Br), −0.7 [3C; Si(*C*H_3_)_3_] ppm.

Thio­phenol (5.83 mmol) was then added to sodium hydride (5.83 mmol) in DMF (5 mL) at 273.15 K and stirred for 10 minutes. The NaSPh solution was added to **2** in DMF (10 mL) at 273.15 K and stirred for 10 minutes. The reaction solution was irradiated with microwaves (150 W, 323.15 K, 1 h) and then quenched with water. The aqueous phase was extracted three times with di­chloro­methane, the combined organic layers were dried over magnesium sulfate and the volatiles were removed. The residue was separated by fractional distillation under reduced pressure. Crystallization from diethyl ether at 243.15 K provided silylated thio­ether ligand **3** as clear and colourless blocks.

^1^H NMR (600 MHz, CDCl_3_) δ = 7.38–7.34 (*m*, 6H; C*H_ortho_*), 7.30–7.23 (*m*, 6H; C*H_meta_*), 7.19–7.15 (*m*, 3H; C*H_para_*), 3.60 (*s*, 2H; OC*H*_2_C), 3.22 (*s*, 6H; SC*H*_2_C), 0.04 [*s*, 9H; Si(C*H*_3_)_3_] ppm.

{^1^H}^13^C NMR (151 MHz, CDCl_3_) δ = 137.2 (3C; *C_ipso_*), 129.8 (3C; *C_ortho_*), 128.9 (3C; *C_meta_*), 126.2 (3C; *C_para_*), 64.1 (1C; O*C*H_2_C), 46.0 (1C; (*C*(CH_2_)_4_), 38.7 (3C; S*C*H_2_C), −0.6 [3C; Si(*C*H_3_)_3_] ppm.

## 6. Refinement

Crystal data, data collection and structure refinement details are summarized in Table 4 ▸. H atoms were positioned geometrically (C—H = 0.95–1.00 Å) and were refined using a riding model, with *U*_iso_(H) = 1.2*U*_eq_(C) for CH_2_ and CH hydrogen atoms and *U*_iso_(H) = 1.5*U*_eq_(C) for CH_3_ hydrogen atoms. Hydrogen atoms H1, H2, H3 and H4 for compound **1** were refined independently. The TMSO group in **3** is disordered with occupancies converging to 50.9 (3)% for O1/Si1/C24–C26 and 49.1 (3)% for O1′/Si1′/C24′–C26′.

## Supplementary Material

Crystal structure: contains datablock(s) 1, 3, global. DOI: 10.1107/S205698902300227X/vm2278sup1.cif

Structure factors: contains datablock(s) 1. DOI: 10.1107/S205698902300227X/vm22781sup2.hkl

Structure factors: contains datablock(s) 3. DOI: 10.1107/S205698902300227X/vm22783sup3.hkl

Supporting information file. DOI: 10.1107/S205698902300227X/vm22781sup4.cml

Supporting information file. DOI: 10.1107/S205698902300227X/vm22783sup5.cml

CCDC references: 2247226, 2247225

Additional supporting information:  crystallographic information; 3D view; checkCIF report

## Figures and Tables

**Table 1:** Selected geometric parameters (Å, °) for **1** [Chem scheme1]

Br1—C3	1.948 (3)	Br8—C14	1.947 (3)
Br2—C4	1.952 (3)	Br9—C15	1.951 (3)
Br3—C5	1.950 (3)	Br10—C18	1.957 (3)
Br4—C8	1.956 (3)	Br11—C19	1.955 (3)
Br5—C9	1.956 (3)	Br12—C20	1.955 (3)
Br6—C10	1.954 (3)	O1—C2	1.432 (3)
Br7—C13	1.951 (3)		
			
C1—C3—Br1	112.99 (18)	C1—C5—Br3	113.21 (19)
C1—C4—Br2	113.48 (18)	O1—C2—C1	111.6 (2)

**Table 2:** Selected geometric parameters (Å, °) for **3** [Chem scheme1]

S1—C7	1.8244 (10)	O1—C23	1.384 (15)
S1—C1	1.7661 (11)	O1′—C23	1.479 (14)
S2—C10	1.7625 (12)	O1—Si1	1.632 (12)
S3—C16	1.8214 (11)	O1′—Si1′	1.672 (13)
S3—C17	1.7742 (13)		
			
C1—S1—C7	105.85 (5)	C23—O1′—Si1′	123.7 (7)
C10—S2—C9	103.65 (5)	C23—O1—Si1	123.6 (7)
C17—S3—C16	104.74 (5)		

**Table 3:** Hydrogen-bond geometry (Å, °) for **1** [Chem scheme1]

*D*—H⋯*A*	*D*—H	H⋯*A*	*D*⋯*A*	*D*—H⋯*A*
O2—H2⋯O3	0.75 (4)	2.04 (4)	2.766 (3)	162 (5)
O4—H4⋯O1^i^	0.75 (5)	2.05 (5)	2.773 (3)	161 (5)
O3—H3⋯O4	0.74 (4)	1.99 (4)	2.729 (3)	175 (4)
O1—H1⋯O2	0.72 (4)	2.00 (4)	2.712 (3)	168 (4)

Symmetry code: (i) 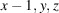 .

**Table 4:** Experimental details

	**1**	**3**
Crystal data		
Chemical formula	4C_5_H_9_Br_3_O	C_26_H_32_OS_3_Si
*M*_r_	1299.35	484.78
Crystal system, space group	Triclinic, *P* 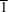	Monoclinic, *C*2/*c*
Temperature (K)	100	100
*a*, *b*, *c* (Å)	8.7763 (2), 9.4409 (3), 21.2951 (6)	27.0415 (12), 6.9329 (3), 27.7963 (14)
α, β, γ (°)	96.762 (1), 92.198 (1), 90.552 (1)	90, 96.939 (2), 90
*V* (Å^3^)	1750.69 (8)	5173.0 (4)
*Z*	2	8
Radiation type	Mo *K*α	Mo *K*α
μ (mm^−1^)	13.75	0.35
Crystal size (mm)	0.19 × 0.14 × 0.1	0.62 × 0.56 × 0.46

Data collection		
Diffractometer	Bruker D8 VENTURE	Bruker D8 VENTURE
Absorption correction	Multi-scan (*SADABS*; Krause *et al.*, 2015 ▸)	Multi-scan (*SADABS*; Krause *et al.*, 2015 ▸)
*T*_min_, *T*_max_	0.337, 0.565	0.671, 0.746
No. of measured, independent and observed [*I* > 2σ(*I*)] reflections	61804, 10208, 8756	40629, 8253, 6927
*R*_int_	0.049	0.035
(sin θ/λ)_max_ (Å^−1^)	0.703	0.726

Refinement		
*R*[*F* ^2^ > 2σ(*F* ^2^)], *wR*(*F* ^2^), *S*	0.027, 0.069, 1.02	0.032, 0.077, 1.03
No. of reflections	10208	8253
No. of parameters	341	340
H-atom treatment	H atoms treated by a mixture of independent and constrained refinement	H atoms treated by a mixture of independent and constrained refinement
Δρ_max_, Δρ_min_ (e Å^−3^)	1.30, −1.02	0.36, −0.24

Computer programs: *SAINT* (Bruker, 2016 ▸, 2018 ▸), *SHELXT* (Sheldrick, 2015*a* ▸), *SHELXL2014/7* (Sheldrick, 2015*b* ▸), *OLEX2* (Dolomanov *et al.*, 2009 ▸), *CrystalExplorer21* (Spackman *et al.*, 2021 ▸), *Mercury* (Macrae *et al.*, 2020 ▸) and *publCIF* (Westrip, 2010 ▸).

## supplementary crystallographic information

### 3-Bromo-2,2-bis(bromomethyl)propan-1-ol (1) Crystal data

**Table:**

4C_5_H_9_Br_3_O	*Z* = 2
*M**_r_* = 1299.35	*F*(000) = 1216
Triclinic, *P*1	*D*_x_ = 2.465 Mg m^−^^3^
*a* = 8.7763 (2) Å	Mo *K*α radiation, λ = 0.71073 Å
*b* = 9.4409 (3) Å	Cell parameters from 7029 reflections
*c* = 21.2951 (6) Å	θ = 2.3–15.9°
α = 96.762 (1)°	µ = 13.75 mm^−^^1^
β = 92.198 (1)°	*T* = 100 K
γ = 90.552 (1)°	Block, clear colourless
*V* = 1750.69 (8) Å^3^	0.19 × 0.14 × 0.1 mm

### 3-Bromo-2,2-bis(bromomethyl)propan-1-ol (1) Data collection

**Table:**

Bruker D8 VENTURE diffractometer	10208 independent reflections
Radiation source: microfocus sealed X-ray tube, Incoatec Iµs	8756 reflections with *I* > 2σ(*I*)
HELIOS mirror optics monochromator	*R*_int_ = 0.049
Detector resolution: 10.4167 pixels mm^-1^	θ_max_ = 30.0°, θ_min_ = 1.9°
ω and φ scans	*h* = −12→12
Absorption correction: multi-scan (SADABS; Krause *et al.*, 2015)	*k* = −13→13
*T*_min_ = 0.337, *T*_max_ = 0.565	*l* = −29→29
61804 measured reflections	

### 3-Bromo-2,2-bis(bromomethyl)propan-1-ol (1) Refinement

**Table:**

Refinement on *F*^2^	Primary atom site location: dual
Least-squares matrix: full	Hydrogen site location: mixed
*R*[*F*^2^ > 2σ(*F*^2^)] = 0.027	H atoms treated by a mixture of independent and constrained refinement
*wR*(*F*^2^) = 0.069	*w* = 1/[σ^2^(*F*_o_^2^) + (0.0285*P*)^2^ + 2.5017*P*] where *P* = (*F*_o_^2^ + 2*F*_c_^2^)/3
*S* = 1.02	(Δ/σ)_max_ = 0.002
10208 reflections	Δρ_max_ = 1.30 e Å^−^^3^
341 parameters	Δρ_min_ = −1.02 e Å^−^^3^
0 restraints	

### 3-Bromo-2,2-bis(bromomethyl)propan-1-ol (1) Special details

**Table:**

Geometry. All esds (except the esd in the dihedral angle between two l.s. planes) are estimated using the full covariance matrix. The cell esds are taken into account individually in the estimation of esds in distances, angles and torsion angles; correlations between esds in cell parameters are only used when they are defined by crystal symmetry. An approximate (isotropic) treatment of cell esds is used for estimating esds involving l.s. planes.

### 3-Bromo-2,2-bis(bromomethyl)propan-1-ol (1) Fractional atomic coordinates and isotropic or equivalent isotropic displacement parameters (Å^2^)

**Table:**

	*x*	*y*	*z*	*U*_iso_*/*U*_eq_	
Br6	0.30125 (3)	0.63039 (3)	0.93269 (2)	0.02037 (6)	
Br4	0.86961 (3)	0.53671 (3)	0.92653 (2)	0.02067 (6)	
Br12	−0.19710 (3)	0.09408 (3)	0.93843 (2)	0.02543 (7)	
Br8	0.16003 (3)	0.66948 (3)	0.52163 (2)	0.02341 (6)	
Br11	0.01625 (3)	−0.18319 (3)	0.81305 (2)	0.02304 (6)	
Br5	0.51228 (3)	0.84879 (3)	0.81175 (2)	0.02386 (7)	
Br10	0.36938 (3)	0.18854 (3)	0.93546 (2)	0.02617 (7)	
Br2	0.65686 (3)	−0.15652 (3)	0.52219 (2)	0.02328 (6)	
Br9	0.57548 (3)	0.49690 (3)	0.59268 (2)	0.02586 (7)	
Br3	1.07850 (3)	0.04690 (3)	0.59305 (2)	0.02689 (7)	
Br7	−0.05124 (3)	0.56946 (3)	0.67027 (2)	0.02622 (7)	
Br1	0.45074 (3)	0.02393 (3)	0.67092 (2)	0.02651 (7)	
O2	0.6078 (3)	0.3698 (2)	0.78090 (11)	0.0242 (5)	
O4	0.1096 (3)	0.2803 (2)	0.78670 (12)	0.0251 (5)	
O3	0.3270 (3)	0.4014 (2)	0.72095 (10)	0.0210 (4)	
O1	0.8317 (3)	0.2138 (2)	0.72320 (10)	0.0204 (4)	
C10	0.4056 (3)	0.5476 (3)	0.85794 (13)	0.0179 (5)	
H10A	0.3892	0.4429	0.8528	0.022*	
H10B	0.3591	0.5846	0.8202	0.022*	
C11	0.2610 (3)	0.5306 (3)	0.63072 (13)	0.0145 (5)	
C1	0.7645 (3)	0.0382 (3)	0.63190 (13)	0.0147 (5)	
C6	0.5773 (3)	0.5793 (3)	0.86066 (13)	0.0156 (5)	
C4	0.7044 (3)	0.0353 (3)	0.56350 (14)	0.0188 (5)	
H4A	0.7816	0.0798	0.5391	0.023*	
H4B	0.6111	0.0934	0.5628	0.023*	
C14	0.2011 (3)	0.4988 (3)	0.56232 (14)	0.0183 (5)	
H14A	0.1059	0.4413	0.5613	0.022*	
H14B	0.2768	0.4408	0.5378	0.022*	
C20	−0.0922 (3)	0.1385 (3)	0.86366 (14)	0.0198 (5)	
H20A	−0.1390	0.0808	0.8259	0.024*	
H20B	−0.1081	0.2403	0.8585	0.024*	
C16	0.0793 (3)	0.1105 (3)	0.86637 (13)	0.0175 (5)	
C9	0.6137 (3)	0.7389 (3)	0.87314 (14)	0.0186 (5)	
H9A	0.7253	0.7537	0.8720	0.022*	
H9B	0.5820	0.7752	0.9161	0.022*	
C8	0.6491 (3)	0.5059 (3)	0.91482 (14)	0.0184 (5)	
H8A	0.6276	0.4021	0.9064	0.022*	
H8B	0.6001	0.5418	0.9546	0.022*	
C7	0.6409 (3)	0.5181 (3)	0.79682 (14)	0.0196 (5)	
H7A	0.5972	0.5710	0.7631	0.024*	
H7B	0.7529	0.5332	0.7986	0.024*	
C13	0.1613 (3)	0.6329 (3)	0.67193 (14)	0.0186 (5)	
H13A	0.1659	0.7283	0.6571	0.022*	
H13B	0.2028	0.6424	0.7161	0.022*	
C3	0.6604 (3)	−0.0418 (3)	0.67253 (14)	0.0189 (5)	
H3A	0.7016	−0.0296	0.7168	0.023*	
H3B	0.6612	−0.1449	0.6571	0.023*	
C17	0.1445 (3)	0.1412 (3)	0.80276 (14)	0.0203 (5)	
H17A	0.2566	0.1309	0.8051	0.024*	
H17B	0.1031	0.0693	0.7687	0.024*	
C18	0.1501 (3)	0.2131 (3)	0.92103 (15)	0.0217 (6)	
H18A	0.0978	0.1992	0.9603	0.026*	
H18B	0.1319	0.3122	0.9121	0.026*	
C15	0.4154 (3)	0.6086 (3)	0.63522 (14)	0.0184 (5)	
H15A	0.4467	0.6330	0.6804	0.022*	
H15B	0.4042	0.6990	0.6163	0.022*	
C12	0.2713 (3)	0.3858 (3)	0.65646 (13)	0.0173 (5)	
H12A	0.1691	0.3397	0.6534	0.021*	
H12B	0.3403	0.3233	0.6303	0.021*	
C2	0.7795 (3)	0.1961 (3)	0.65817 (13)	0.0174 (5)	
H2A	0.8522	0.2441	0.6329	0.021*	
H2B	0.6792	0.2421	0.6542	0.021*	
C19	0.1148 (3)	−0.0427 (3)	0.87699 (14)	0.0199 (5)	
H19A	0.0808	−0.0598	0.9193	0.024*	
H19B	0.2265	−0.0558	0.8766	0.024*	
C5	0.9158 (3)	−0.0390 (3)	0.63634 (15)	0.0202 (5)	
H5A	0.9009	−0.1395	0.6178	0.024*	
H5B	0.9472	−0.0387	0.6815	0.024*	
H2	0.535 (5)	0.361 (5)	0.761 (2)	0.034 (12)*	
H4	0.036 (5)	0.280 (5)	0.768 (2)	0.046 (14)*	
H3	0.264 (5)	0.369 (5)	0.737 (2)	0.031 (11)*	
H1	0.766 (5)	0.245 (4)	0.7388 (19)	0.027 (11)*	

### 3-Bromo-2,2-bis(bromomethyl)propan-1-ol (1) Atomic displacement parameters (Å^2^)

**Table:**

	*U*^11^	*U*^22^	*U*^33^	*U*^12^	*U*^13^	*U*^23^
Br6	0.01720 (12)	0.02772 (14)	0.01664 (13)	0.00405 (10)	0.00425 (10)	0.00301 (10)
Br4	0.01508 (12)	0.02315 (13)	0.02331 (14)	0.00189 (10)	−0.00250 (10)	0.00171 (11)
Br12	0.02147 (14)	0.03204 (15)	0.02229 (15)	−0.00074 (11)	0.00614 (11)	−0.00048 (12)
Br8	0.02755 (15)	0.02032 (13)	0.02272 (15)	0.00141 (11)	−0.00450 (11)	0.00575 (11)
Br11	0.02468 (14)	0.01963 (13)	0.02347 (15)	−0.00413 (10)	0.00482 (11)	−0.00391 (11)
Br5	0.02543 (14)	0.02445 (14)	0.02381 (15)	0.00620 (11)	0.00529 (11)	0.00985 (11)
Br10	0.01955 (14)	0.02889 (15)	0.02851 (16)	−0.00311 (11)	−0.00459 (11)	−0.00105 (12)
Br2	0.02747 (15)	0.01877 (13)	0.02193 (15)	−0.00285 (10)	−0.00348 (11)	−0.00290 (10)
Br9	0.01620 (13)	0.02863 (15)	0.03546 (18)	0.00502 (10)	0.00516 (12)	0.01344 (13)
Br3	0.01618 (13)	0.02337 (14)	0.03854 (18)	−0.00393 (10)	0.00650 (12)	−0.00860 (12)
Br7	0.01773 (13)	0.02374 (14)	0.03600 (18)	−0.00242 (10)	0.00665 (12)	−0.00308 (12)
Br1	0.01738 (13)	0.02924 (15)	0.03493 (18)	0.00410 (11)	0.00650 (12)	0.01025 (13)
O2	0.0205 (11)	0.0261 (11)	0.0239 (12)	0.0031 (8)	−0.0009 (9)	−0.0056 (9)
O4	0.0208 (11)	0.0281 (11)	0.0277 (12)	−0.0036 (9)	0.0004 (9)	0.0091 (9)
O3	0.0220 (10)	0.0240 (10)	0.0173 (10)	−0.0044 (8)	−0.0020 (8)	0.0042 (8)
O1	0.0198 (10)	0.0219 (10)	0.0181 (10)	0.0044 (8)	−0.0016 (8)	−0.0027 (8)
C10	0.0176 (12)	0.0222 (13)	0.0136 (13)	0.0003 (10)	0.0011 (10)	0.0003 (10)
C11	0.0145 (11)	0.0134 (11)	0.0151 (12)	−0.0010 (9)	−0.0008 (9)	0.0008 (9)
C1	0.0159 (12)	0.0124 (11)	0.0160 (13)	0.0000 (9)	0.0002 (10)	0.0026 (9)
C6	0.0142 (11)	0.0185 (12)	0.0140 (12)	−0.0006 (9)	0.0017 (9)	0.0013 (9)
C4	0.0217 (13)	0.0135 (11)	0.0208 (14)	−0.0008 (10)	−0.0018 (11)	0.0011 (10)
C14	0.0203 (13)	0.0154 (12)	0.0182 (13)	−0.0004 (9)	−0.0040 (10)	0.0002 (10)
C20	0.0199 (13)	0.0228 (13)	0.0164 (13)	0.0013 (10)	0.0017 (10)	0.0006 (10)
C16	0.0172 (12)	0.0174 (12)	0.0173 (13)	−0.0002 (9)	0.0018 (10)	−0.0012 (10)
C9	0.0187 (12)	0.0199 (12)	0.0184 (13)	0.0012 (10)	0.0015 (10)	0.0063 (10)
C8	0.0135 (12)	0.0228 (13)	0.0191 (13)	0.0005 (10)	−0.0006 (10)	0.0029 (10)
C7	0.0178 (13)	0.0242 (13)	0.0167 (13)	−0.0002 (10)	0.0019 (10)	0.0018 (10)
C13	0.0168 (12)	0.0168 (12)	0.0210 (14)	−0.0016 (9)	−0.0004 (10)	−0.0026 (10)
C3	0.0170 (12)	0.0190 (12)	0.0214 (14)	0.0009 (10)	−0.0009 (10)	0.0053 (10)
C17	0.0205 (13)	0.0222 (13)	0.0181 (14)	−0.0016 (10)	0.0011 (10)	0.0014 (10)
C18	0.0179 (13)	0.0246 (14)	0.0207 (14)	−0.0008 (10)	−0.0011 (11)	−0.0040 (11)
C15	0.0161 (12)	0.0152 (11)	0.0238 (14)	−0.0021 (9)	−0.0003 (10)	0.0026 (10)
C12	0.0198 (13)	0.0145 (11)	0.0171 (13)	−0.0008 (9)	0.0003 (10)	−0.0001 (10)
C2	0.0203 (13)	0.0144 (11)	0.0170 (13)	0.0019 (9)	0.0007 (10)	−0.0005 (10)
C19	0.0215 (13)	0.0184 (12)	0.0186 (14)	0.0002 (10)	−0.0017 (11)	−0.0025 (10)
C5	0.0164 (12)	0.0175 (12)	0.0259 (15)	0.0025 (10)	−0.0009 (11)	0.0004 (11)

### 3-Bromo-2,2-bis(bromomethyl)propan-1-ol (1) Geometric parameters (Å, º)

**Table:**

Br1—C3	1.948 (3)	C4—H4A	0.9900
Br2—C4	1.952 (3)	C4—H4B	0.9900
Br3—C5	1.950 (3)	C14—H14A	0.9900
Br4—C8	1.956 (3)	C14—H14B	0.9900
Br5—C9	1.956 (3)	C20—H20A	0.9900
Br6—C10	1.954 (3)	C20—H20B	0.9900
Br7—C13	1.951 (3)	C20—C16	1.531 (4)
Br8—C14	1.947 (3)	C16—C17	1.548 (4)
Br9—C15	1.951 (3)	C16—C18	1.532 (4)
Br10—C18	1.957 (3)	C16—C19	1.523 (4)
Br11—C19	1.955 (3)	C9—H9A	0.9900
Br12—C20	1.955 (3)	C9—H9B	0.9900
O2—C7	1.426 (4)	C8—H8A	0.9900
O2—H2	0.75 (4)	C8—H8B	0.9900
O4—C17	1.428 (4)	C7—H7A	0.9900
O4—H4	0.75 (5)	C7—H7B	0.9900
O3—C12	1.431 (3)	C13—H13A	0.9900
O3—H3	0.74 (4)	C13—H13B	0.9900
O1—C2	1.432 (3)	C3—H3A	0.9900
O1—H1	0.72 (4)	C3—H3B	0.9900
C10—H10A	0.9900	C17—H17A	0.9900
C10—H10B	0.9900	C17—H17B	0.9900
C10—C6	1.531 (4)	C18—H18A	0.9900
C11—C14	1.524 (4)	C18—H18B	0.9900
C11—C13	1.531 (4)	C15—H15A	0.9900
C11—C15	1.530 (4)	C15—H15B	0.9900
C11—C12	1.534 (4)	C12—H12A	0.9900
C1—C4	1.527 (4)	C12—H12B	0.9900
C1—C3	1.535 (4)	C2—H2A	0.9900
C1—C2	1.532 (4)	C2—H2B	0.9900
C1—C5	1.526 (4)	C19—H19A	0.9900
C6—C9	1.528 (4)	C19—H19B	0.9900
C6—C8	1.532 (4)	C5—H5A	0.9900
C6—C7	1.540 (4)	C5—H5B	0.9900
			
C7—O2—H2	109 (3)	H8A—C8—H8B	107.7
C17—O4—H4	111 (4)	O2—C7—C6	113.0 (2)
C12—O3—H3	102 (3)	O2—C7—H7A	109.0
C2—O1—H1	102 (3)	O2—C7—H7B	109.0
Br6—C10—H10A	108.8	C6—C7—H7A	109.0
Br6—C10—H10B	108.8	C6—C7—H7B	109.0
H10A—C10—H10B	107.7	H7A—C7—H7B	107.8
C6—C10—Br6	113.95 (19)	Br7—C13—H13A	109.0
C6—C10—H10A	108.8	Br7—C13—H13B	109.0
C6—C10—H10B	108.8	C11—C13—Br7	112.91 (18)
C14—C11—C13	113.7 (2)	C11—C13—H13A	109.0
C14—C11—C15	111.6 (2)	C11—C13—H13B	109.0
C14—C11—C12	105.9 (2)	H13A—C13—H13B	107.8
C13—C11—C12	110.9 (2)	Br1—C3—H3A	109.0
C15—C11—C13	103.0 (2)	Br1—C3—H3B	109.0
C15—C11—C12	111.9 (2)	C1—C3—Br1	112.99 (18)
C4—C1—C3	113.2 (2)	C1—C4—Br2	113.48 (18)
C4—C1—C2	105.9 (2)	C1—C3—H3A	109.0
C2—C1—C3	110.8 (2)	C1—C3—H3B	109.0
C5—C1—C4	111.9 (2)	H3A—C3—H3B	107.8
C5—C1—C3	103.2 (2)	O4—C17—C16	113.3 (2)
C5—C1—C2	112.0 (2)	O4—C17—H17A	108.9
C10—C6—C8	107.7 (2)	O4—C17—H17B	108.9
C10—C6—C7	107.9 (2)	C16—C17—H17A	108.9
C9—C6—C10	112.4 (2)	C16—C17—H17B	108.9
C9—C6—C8	108.6 (2)	H17A—C17—H17B	107.7
C9—C6—C7	109.6 (2)	Br10—C18—H18A	108.8
C8—C6—C7	110.7 (2)	Br10—C18—H18B	108.8
Br2—C4—H4A	108.9	C16—C18—Br10	113.95 (19)
Br2—C4—H4B	108.9	C16—C18—H18A	108.8
C1—C4—H4A	108.9	C16—C18—H18B	108.8
C1—C4—H4B	108.9	H18A—C18—H18B	107.7
H4A—C4—H4B	107.7	Br9—C15—H15A	108.9
Br8—C14—H14A	108.9	Br9—C15—H15B	108.9
Br8—C14—H14B	108.9	C11—C15—Br9	113.19 (18)
C11—C14—Br8	113.49 (18)	C11—C15—H15A	108.9
C11—C14—H14A	108.9	C11—C15—H15B	108.9
C11—C14—H14B	108.9	H15A—C15—H15B	107.8
H14A—C14—H14B	107.7	O3—C12—C11	111.4 (2)
Br12—C20—H20A	108.8	O3—C12—H12A	109.4
Br12—C20—H20B	108.8	O3—C12—H12B	109.4
H20A—C20—H20B	107.7	C11—C12—H12A	109.4
C16—C20—Br12	114.0 (2)	C11—C12—H12B	109.4
C16—C20—H20A	108.8	H12A—C12—H12B	108.0
C16—C20—H20B	108.8	O1—C2—H2A	109.3
C20—C16—C17	108.1 (2)	O1—C2—H2B	109.3
C20—C16—C18	107.3 (2)	C1—C2—H2A	109.3
C18—C16—C17	110.4 (2)	C1—C2—H2B	109.3
C19—C16—C20	112.4 (2)	H2A—C2—H2B	108.0
C19—C16—C17	109.0 (2)	Br11—C19—H19A	109.0
C19—C16—C18	109.7 (2)	Br11—C19—H19B	109.0
Br5—C9—H9A	109.0	C16—C19—Br11	112.9 (2)
Br5—C9—H9B	109.0	C16—C19—H19A	109.0
C6—C9—Br5	113.0 (2)	C16—C19—H19B	109.0
C6—C9—H9A	109.0	H19A—C19—H19B	107.8
C6—C9—H9B	109.0	Br3—C5—H5A	108.9
H9A—C9—H9B	107.8	Br3—C5—H5B	108.9
Br4—C8—H8A	108.8	C1—C5—Br3	113.21 (19)
Br4—C8—H8B	108.8	O1—C2—C1	111.6 (2)
C6—C8—Br4	113.72 (19)	C1—C5—H5A	108.9
C6—C8—H8A	108.8	C1—C5—H5B	108.9
C6—C8—H8B	108.8	H5A—C5—H5B	107.7
			
Br6—C10—C6—C9	−54.5 (3)	C13—C11—C14—Br8	52.6 (3)
Br6—C10—C6—C8	65.0 (3)	C13—C11—C15—Br9	175.11 (19)
Br6—C10—C6—C7	−175.47 (18)	C13—C11—C12—O3	−56.3 (3)
Br12—C20—C16—C17	176.50 (18)	C3—C1—C4—Br2	−52.8 (3)
Br12—C20—C16—C18	−64.5 (3)	C3—C1—C2—O1	55.4 (3)
Br12—C20—C16—C19	56.1 (3)	C3—C1—C5—Br3	−176.37 (18)
C10—C6—C9—Br5	−55.4 (3)	C17—C16—C18—Br10	−65.2 (3)
C10—C6—C8—Br4	−179.21 (18)	C17—C16—C19—Br11	−63.5 (3)
C10—C6—C7—O2	−55.7 (3)	C18—C16—C17—O4	−62.8 (3)
C4—C1—C3—Br1	−54.4 (3)	C18—C16—C19—Br11	175.64 (19)
C4—C1—C2—O1	178.5 (2)	C15—C11—C14—Br8	−63.4 (3)
C4—C1—C5—Br3	61.6 (3)	C15—C11—C13—Br7	175.29 (18)
C14—C11—C13—Br7	54.3 (3)	C15—C11—C12—O3	58.1 (3)
C14—C11—C15—Br9	−62.5 (3)	C12—C11—C14—Br8	174.59 (18)
C14—C11—C12—O3	179.9 (2)	C12—C11—C13—Br7	−64.9 (3)
C20—C16—C17—O4	54.2 (3)	C12—C11—C15—Br9	56.0 (3)
C20—C16—C18—Br10	177.22 (19)	C2—C1—C4—Br2	−174.38 (18)
C20—C16—C19—Br11	56.4 (3)	C2—C1—C3—Br1	64.4 (3)
C9—C6—C8—Br4	−57.3 (3)	C2—C1—C5—Br3	−57.1 (3)
C9—C6—C7—O2	−178.4 (2)	C19—C16—C17—O4	176.7 (2)
C8—C6—C9—Br5	−174.45 (17)	C19—C16—C18—Br10	54.8 (3)
C8—C6—C7—O2	61.9 (3)	C5—C1—C4—Br2	63.4 (3)
C7—C6—C9—Br5	64.5 (3)	C5—C1—C3—Br1	−175.56 (18)
C7—C6—C8—Br4	63.0 (3)	C5—C1—C2—O1	−59.3 (3)

### 3-Bromo-2,2-bis(bromomethyl)propan-1-ol (1) Hydrogen-bond geometry (Å, º)

**Table:**

*D*—H···*A*	*D*—H	H···*A*	*D*···*A*	*D*—H···*A*
O2—H2···O3	0.75 (4)	2.04 (4)	2.766 (3)	162 (5)
O4—H4···O1^i^	0.75 (5)	2.05 (5)	2.773 (3)	161 (5)
O3—H3···O4	0.74 (4)	1.99 (4)	2.729 (3)	175 (4)
O1—H1···O2	0.72 (4)	2.00 (4)	2.712 (3)	168 (4)

Symmetry code: (i) *x*−1, *y*, *z*.

### Trimethyl({tris[(phenylsulfanyl)methyl]silyl}methoxy)silane (3) Crystal data

**Table:**

C_26_H_32_OS_3_Si	*F*(000) = 2064
*M**_r_* = 484.78	*D*_x_ = 1.245 Mg m^−^^3^
Monoclinic, *C*2/*c*	Mo *K*α radiation, λ = 0.71073 Å
*a* = 27.0415 (12) Å	Cell parameters from 9874 reflections
*b* = 6.9329 (3) Å	θ = 2.2–31.0°
*c* = 27.7963 (14) Å	µ = 0.35 mm^−^^1^
β = 96.939 (2)°	*T* = 100 K
*V* = 5173.0 (4) Å^3^	Block, clear colourless
*Z* = 8	0.62 × 0.56 × 0.46 mm

### Trimethyl({tris[(phenylsulfanyl)methyl]silyl}methoxy)silane (3) Data collection

**Table:**

Bruker D8 VENTURE diffractometer	8253 independent reflections
Radiation source: microfocus sealed X-ray tube, Incoatec Iµs	6927 reflections with *I* > 2σ(*I*)
HELIOS mirror optics monochromator	*R*_int_ = 0.035
Detector resolution: 10.4167 pixels mm^-1^	θ_max_ = 31.1°, θ_min_ = 2.2°
φ and ω scans	*h* = −39→39
Absorption correction: multi-scan (SADABS; Krause *et al.*, 2015)	*k* = −10→10
*T*_min_ = 0.671, *T*_max_ = 0.746	*l* = −40→40
40629 measured reflections	

### Trimethyl({tris[(phenylsulfanyl)methyl]silyl}methoxy)silane (3) Refinement

**Table:**

Refinement on *F*^2^	Primary atom site location: dual
Least-squares matrix: full	Hydrogen site location: mixed
*R*[*F*^2^ > 2σ(*F*^2^)] = 0.032	H atoms treated by a mixture of independent and constrained refinement
*wR*(*F*^2^) = 0.077	*w* = 1/[σ^2^(*F*_o_^2^) + (0.0273*P*)^2^ + 3.6217*P*] where *P* = (*F*_o_^2^ + 2*F*_c_^2^)/3
*S* = 1.03	(Δ/σ)_max_ = 0.003
8253 reflections	Δρ_max_ = 0.36 e Å^−^^3^
340 parameters	Δρ_min_ = −0.24 e Å^−^^3^
0 restraints	

### Trimethyl({tris[(phenylsulfanyl)methyl]silyl}methoxy)silane (3) Special details

**Table:**

Geometry. All esds (except the esd in the dihedral angle between two l.s. planes) are estimated using the full covariance matrix. The cell esds are taken into account individually in the estimation of esds in distances, angles and torsion angles; correlations between esds in cell parameters are only used when they are defined by crystal symmetry. An approximate (isotropic) treatment of cell esds is used for estimating esds involving l.s. planes.

### Trimethyl({tris[(phenylsulfanyl)methyl]silyl}methoxy)silane (3) Fractional atomic coordinates and isotropic or equivalent isotropic displacement parameters (Å^2^)

**Table:**

	*x*	*y*	*z*	*U*_iso_*/*U*_eq_	Occ. (<1)
S1	0.29397 (2)	0.49136 (4)	0.53826 (2)	0.01817 (6)	
S2	0.20238 (2)	0.78150 (4)	0.58269 (2)	0.02262 (6)	
S3	0.31216 (2)	1.14605 (4)	0.66285 (2)	0.02538 (7)	
C8	0.30483 (4)	0.80744 (14)	0.60358 (4)	0.01565 (18)	
C7	0.30656 (4)	0.74642 (14)	0.55036 (4)	0.01713 (18)	
H7A	0.3399	0.7775	0.5412	0.021*	
H7B	0.2819	0.8238	0.5294	0.021*	
C9	0.25831 (4)	0.72995 (14)	0.62389 (4)	0.01716 (18)	
H9A	0.2556	0.7904	0.6557	0.021*	
H9B	0.2615	0.5889	0.6288	0.021*	
C16	0.30594 (4)	1.02993 (15)	0.60364 (4)	0.0201 (2)	
H16A	0.2749	1.0768	0.5848	0.024*	
H16B	0.3340	1.0724	0.5864	0.024*	
C1	0.34951 (4)	0.39457 (15)	0.52076 (4)	0.0198 (2)	
C23	0.34988 (4)	0.72598 (16)	0.63568 (4)	0.01953 (19)	
C15	0.16005 (4)	0.70047 (17)	0.66701 (4)	0.0246 (2)	
H15	0.1914	0.6503	0.6805	0.029*	
C2	0.34409 (5)	0.23215 (16)	0.49074 (4)	0.0250 (2)	
H2	0.3117	0.1857	0.4793	0.030*	
C6	0.39722 (4)	0.46279 (18)	0.53680 (4)	0.0277 (2)	
H6	0.4014	0.5728	0.5573	0.033*	
C17	0.25090 (5)	1.14867 (15)	0.67960 (4)	0.0255 (2)	
C10	0.15440 (4)	0.77326 (17)	0.62029 (4)	0.0230 (2)	
C22	0.24394 (5)	1.07987 (17)	0.72557 (4)	0.0300 (3)	
H22	0.2712	1.0258	0.7459	0.036*	
C18	0.21056 (5)	1.22418 (17)	0.64960 (5)	0.0307 (3)	
H18	0.2150	1.2712	0.6183	0.037*	
C14	0.12017 (5)	0.7002 (2)	0.69433 (5)	0.0330 (3)	
H14	0.1245	0.6515	0.7265	0.040*	
C3	0.38577 (5)	0.13854 (18)	0.47757 (5)	0.0332 (3)	
H3	0.3818	0.0272	0.4576	0.040*	
C21	0.19743 (6)	1.09037 (19)	0.74155 (5)	0.0375 (3)	
H21	0.1930	1.0455	0.7730	0.045*	
C5	0.43855 (5)	0.3689 (2)	0.52267 (5)	0.0371 (3)	
H5	0.4710	0.4167	0.5332	0.044*	
C11	0.10797 (4)	0.8453 (2)	0.60058 (5)	0.0355 (3)	
H11	0.1036	0.8962	0.5686	0.043*	
C4	0.43306 (5)	0.2064 (2)	0.49342 (5)	0.0377 (3)	
H4	0.4615	0.1422	0.4843	0.045*	
C20	0.15751 (6)	1.16595 (19)	0.71180 (6)	0.0414 (3)	
H20	0.1257	1.1738	0.7229	0.050*	
C19	0.16397 (5)	1.23047 (19)	0.66560 (6)	0.0384 (3)	
H19	0.1363	1.2792	0.6449	0.046*	
C13	0.07437 (5)	0.7706 (3)	0.67479 (6)	0.0442 (4)	
H13	0.0471	0.7701	0.6934	0.053*	
C12	0.06846 (5)	0.8419 (3)	0.62797 (6)	0.0482 (4)	
H12	0.0368	0.8892	0.6144	0.058*	
Si1'	0.44785 (2)	0.8553 (2)	0.64956 (3)	0.0190 (2)	0.491 (3)
Si1	0.44762 (2)	0.7677 (2)	0.65917 (3)	0.0212 (2)	0.509 (3)
C26'	0.49264 (14)	0.8897 (5)	0.60512 (14)	0.0309 (7)	0.491 (3)
H26A	0.4950	0.7705	0.5866	0.046*	0.491 (3)
H26B	0.5254	0.9218	0.6222	0.046*	0.491 (3)
H26C	0.4812	0.9949	0.5830	0.046*	0.491 (3)
C25	0.45259 (9)	0.5178 (4)	0.68366 (9)	0.0302 (6)	0.509 (3)
H25A	0.4263	0.4959	0.7044	0.045*	0.509 (3)
H25B	0.4852	0.5003	0.7027	0.045*	0.509 (3)
H25C	0.4489	0.4254	0.6568	0.045*	0.509 (3)
C26	0.49628 (13)	0.8198 (6)	0.61941 (14)	0.0355 (7)	0.509 (3)
H26D	0.4953	0.7212	0.5941	0.053*	0.509 (3)
H26E	0.5291	0.8190	0.6387	0.053*	0.509 (3)
H26F	0.4902	0.9469	0.6044	0.053*	0.509 (3)
C24	0.44964 (10)	0.9441 (4)	0.70963 (9)	0.0321 (6)	0.509 (3)
H24A	0.4819	0.9348	0.7298	0.048*	0.509 (3)
H24B	0.4228	0.9157	0.7293	0.048*	0.509 (3)
H24C	0.4453	1.0748	0.6964	0.048*	0.509 (3)
C24'	0.44206 (10)	1.0777 (4)	0.68546 (11)	0.0341 (7)	0.491 (3)
H24D	0.4307	1.1841	0.6637	0.051*	0.491 (3)
H24E	0.4745	1.1101	0.7032	0.051*	0.491 (3)
H24F	0.4179	1.0565	0.7085	0.051*	0.491 (3)
C25'	0.46508 (10)	0.6452 (4)	0.68981 (11)	0.0346 (7)	0.491 (3)
H25D	0.4425	0.6380	0.7148	0.052*	0.491 (3)
H25E	0.4994	0.6607	0.7053	0.052*	0.491 (3)
H25F	0.4625	0.5264	0.6706	0.052*	0.491 (3)
O1'	0.3933 (5)	0.813 (2)	0.6162 (5)	0.0183 (11)	0.491 (3)
O1	0.3954 (5)	0.788 (2)	0.6234 (5)	0.0193 (11)	0.509 (3)
H23A	0.3479 (5)	0.7632 (18)	0.6703 (5)	0.021 (3)*	
H23B	0.3497 (5)	0.5849 (19)	0.6332 (5)	0.019 (3)*	

### Trimethyl({tris[(phenylsulfanyl)methyl]silyl}methoxy)silane (3) Atomic displacement parameters (Å^2^)

**Table:**

	*U*^11^	*U*^22^	*U*^33^	*U*^12^	*U*^13^	*U*^23^
S1	0.01888 (11)	0.01749 (11)	0.01825 (12)	−0.00061 (9)	0.00277 (9)	−0.00230 (9)
S2	0.01735 (11)	0.03543 (15)	0.01493 (12)	0.00120 (10)	0.00136 (9)	−0.00211 (10)
S3	0.03324 (15)	0.02129 (12)	0.02207 (13)	−0.00472 (11)	0.00528 (11)	−0.00615 (10)
C8	0.0167 (4)	0.0162 (4)	0.0140 (4)	−0.0005 (3)	0.0020 (3)	0.0005 (3)
C7	0.0191 (4)	0.0170 (4)	0.0153 (4)	−0.0002 (4)	0.0022 (3)	−0.0002 (3)
C9	0.0182 (4)	0.0189 (4)	0.0145 (4)	−0.0010 (4)	0.0025 (3)	−0.0002 (3)
C16	0.0264 (5)	0.0174 (4)	0.0174 (5)	−0.0018 (4)	0.0058 (4)	−0.0006 (4)
C1	0.0234 (5)	0.0207 (5)	0.0156 (5)	0.0035 (4)	0.0030 (4)	−0.0004 (4)
C23	0.0178 (4)	0.0226 (5)	0.0177 (5)	0.0000 (4)	0.0004 (4)	−0.0010 (4)
C15	0.0225 (5)	0.0276 (5)	0.0244 (5)	−0.0024 (4)	0.0061 (4)	−0.0026 (4)
C2	0.0308 (6)	0.0227 (5)	0.0225 (5)	−0.0004 (4)	0.0063 (4)	−0.0040 (4)
C6	0.0228 (5)	0.0340 (6)	0.0255 (6)	0.0040 (5)	0.0001 (4)	−0.0091 (5)
C17	0.0377 (6)	0.0157 (4)	0.0246 (5)	−0.0008 (4)	0.0103 (5)	−0.0049 (4)
C10	0.0180 (5)	0.0295 (5)	0.0219 (5)	−0.0023 (4)	0.0039 (4)	−0.0065 (4)
C22	0.0471 (7)	0.0225 (5)	0.0218 (6)	−0.0035 (5)	0.0095 (5)	−0.0062 (4)
C18	0.0409 (7)	0.0192 (5)	0.0336 (7)	0.0043 (5)	0.0113 (5)	0.0003 (4)
C14	0.0299 (6)	0.0432 (7)	0.0281 (6)	−0.0080 (5)	0.0129 (5)	−0.0038 (5)
C3	0.0426 (7)	0.0280 (6)	0.0306 (6)	0.0054 (5)	0.0102 (5)	−0.0072 (5)
C21	0.0580 (9)	0.0275 (6)	0.0313 (7)	−0.0067 (6)	0.0229 (6)	−0.0097 (5)
C5	0.0230 (6)	0.0510 (8)	0.0368 (7)	0.0078 (6)	0.0021 (5)	−0.0108 (6)
C11	0.0206 (5)	0.0593 (9)	0.0259 (6)	0.0044 (6)	0.0005 (4)	−0.0050 (6)
C4	0.0347 (7)	0.0446 (7)	0.0347 (7)	0.0163 (6)	0.0080 (5)	−0.0067 (6)
C20	0.0495 (8)	0.0259 (6)	0.0546 (9)	−0.0009 (6)	0.0302 (7)	−0.0126 (6)
C19	0.0403 (7)	0.0246 (6)	0.0522 (9)	0.0087 (5)	0.0130 (6)	−0.0040 (6)
C13	0.0212 (6)	0.0742 (11)	0.0396 (8)	−0.0059 (6)	0.0136 (5)	−0.0133 (7)
C12	0.0183 (6)	0.0866 (12)	0.0394 (8)	0.0053 (7)	0.0028 (5)	−0.0113 (8)
Si1'	0.0157 (3)	0.0224 (5)	0.0190 (3)	−0.0008 (3)	0.0016 (2)	−0.0026 (3)
Si1	0.0166 (3)	0.0259 (6)	0.0208 (3)	−0.0004 (3)	0.0014 (2)	−0.0031 (3)
C26'	0.0251 (13)	0.0358 (18)	0.0338 (18)	−0.0041 (13)	0.0118 (12)	−0.0020 (13)
C25	0.0293 (12)	0.0294 (13)	0.0315 (13)	0.0064 (10)	0.0017 (10)	0.0022 (10)
C26	0.0256 (13)	0.042 (2)	0.041 (2)	−0.0075 (14)	0.0115 (13)	−0.0063 (14)
C24	0.0323 (12)	0.0354 (13)	0.0272 (12)	−0.0013 (10)	−0.0024 (9)	−0.0072 (10)
C24'	0.0216 (11)	0.0366 (14)	0.0437 (16)	−0.0031 (10)	0.0021 (10)	−0.0184 (12)
C25'	0.0277 (12)	0.0378 (16)	0.0373 (15)	0.0041 (11)	−0.0003 (10)	0.0125 (12)
O1'	0.0127 (13)	0.025 (3)	0.016 (3)	−0.0020 (18)	−0.0013 (17)	0.0021 (18)
O1	0.0191 (16)	0.022 (3)	0.016 (3)	−0.0006 (15)	0.0003 (18)	0.0033 (17)

### Trimethyl({tris[(phenylsulfanyl)methyl]silyl}methoxy)silane (3) Geometric parameters (Å, º)

**Table:**

S1—C7	1.8244 (10)	C21—H21	0.9500
S1—C1	1.7661 (11)	C21—C20	1.381 (2)
S2—C9	1.8188 (10)	C5—H5	0.9500
S2—C10	1.7625 (12)	C5—C4	1.3864 (19)
S3—C16	1.8214 (11)	C11—H11	0.9500
S3—C17	1.7742 (13)	C11—C12	1.3850 (19)
C8—C7	1.5446 (14)	C4—H4	0.9500
C8—C9	1.5367 (14)	C20—H20	0.9500
C8—C16	1.5428 (14)	C20—C19	1.391 (2)
C8—C23	1.5286 (14)	C19—H19	0.9500
C7—H7A	0.9900	C13—H13	0.9500
C7—H7B	0.9900	C13—C12	1.383 (2)
C9—H9A	0.9900	C12—H12	0.9500
C9—H9B	0.9900	Si1'—C26'	1.847 (4)
C16—H16A	0.9900	Si1'—C24'	1.853 (3)
C16—H16B	0.9900	Si1'—C25'	1.861 (3)
C1—C2	1.3986 (15)	O1—Si1	1.632 (12)
C1—C6	1.3957 (15)	O1'—Si1'	1.672 (13)
O1—C23	1.384 (15)	Si1—C25	1.861 (3)
O1'—C23	1.479 (14)	Si1—C26	1.853 (4)
C23—H23A	1.004 (14)	Si1—C24	1.856 (3)
C23—H23B	0.981 (13)	C26'—H26A	0.9800
C15—H15	0.9500	C26'—H26B	0.9800
C15—C10	1.3845 (16)	C26'—H26C	0.9800
C15—C14	1.3918 (16)	C25—H25A	0.9800
C2—H2	0.9500	C25—H25B	0.9800
C2—C3	1.3875 (17)	C25—H25C	0.9800
C6—H6	0.9500	C26—H26D	0.9800
C6—C5	1.3905 (17)	C26—H26E	0.9800
C17—C22	1.3977 (17)	C26—H26F	0.9800
C17—C18	1.3925 (18)	C24—H24A	0.9800
C10—C11	1.3998 (16)	C24—H24B	0.9800
C22—H22	0.9500	C24—H24C	0.9800
C22—C21	1.3856 (19)	C24'—H24D	0.9800
C18—H18	0.9500	C24'—H24E	0.9800
C18—C19	1.3865 (19)	C24'—H24F	0.9800
C14—H14	0.9500	C25'—H25D	0.9800
C14—C13	1.380 (2)	C25'—H25E	0.9800
C3—H3	0.9500	C25'—H25F	0.9800
C3—C4	1.384 (2)		
			
C1—S1—C7	105.85 (5)	C4—C5—C6	120.88 (12)
C10—S2—C9	103.65 (5)	C4—C5—H5	119.6
C17—S3—C16	104.74 (5)	C10—C11—H11	120.2
C9—C8—C7	112.14 (8)	C12—C11—C10	119.60 (13)
C9—C8—C16	111.46 (8)	C12—C11—H11	120.2
C16—C8—C7	105.78 (8)	C3—C4—C5	119.50 (12)
C23—C8—C7	110.11 (8)	C3—C4—H4	120.3
C23—C8—C9	106.65 (8)	C5—C4—H4	120.3
C23—C8—C16	110.77 (8)	C21—C20—H20	120.1
S1—C7—H7A	108.6	C21—C20—C19	119.90 (13)
S1—C7—H7B	108.6	C19—C20—H20	120.1
C8—C7—S1	114.55 (7)	C18—C19—C20	120.43 (14)
C8—C7—H7A	108.6	C18—C19—H19	119.8
C8—C7—H7B	108.6	C20—C19—H19	119.8
H7A—C7—H7B	107.6	C14—C13—H13	120.2
S2—C9—H9A	109.5	C14—C13—C12	119.53 (12)
S2—C9—H9B	109.5	C12—C13—H13	120.2
C8—C9—S2	110.82 (7)	C11—C12—H12	119.5
C8—C9—H9A	109.5	C13—C12—C11	120.94 (13)
C8—C9—H9B	109.5	C13—C12—H12	119.5
H9A—C9—H9B	108.1	C26'—Si1'—C24'	110.96 (16)
S3—C16—H16A	108.2	C26'—Si1'—C25'	111.49 (15)
S3—C16—H16B	108.2	C24'—Si1'—C25'	111.01 (15)
C8—C16—S3	116.27 (7)	O1'—Si1'—C26'	104.9 (5)
C8—C16—H16A	108.2	O1'—Si1'—C24'	108.7 (6)
C8—C16—H16B	108.2	O1'—Si1'—C25'	109.6 (4)
H16A—C16—H16B	107.4	C26—Si1—C25	112.00 (15)
C2—C1—S1	116.04 (8)	C26—Si1—C24	111.33 (15)
C6—C1—S1	124.56 (8)	C24—Si1—C25	110.04 (13)
C6—C1—C2	119.31 (10)	O1—Si1—C25	108.8 (5)
C8—C23—H23A	109.5 (7)	O1—Si1—C26	104.2 (5)
C8—C23—H23B	109.2 (7)	O1—Si1—C24	110.4 (6)
O1'—C23—C8	104.4 (4)	Si1'—C26'—H26A	109.5
O1'—C23—H23A	112.6 (10)	Si1'—C26'—H26B	109.5
O1'—C23—H23B	112.1 (10)	Si1'—C26'—H26C	109.5
O1—C23—C8	114.3 (4)	H26A—C26'—H26B	109.5
O1—C23—H23A	108.0 (10)	H26A—C26'—H26C	109.5
O1—C23—H23B	106.8 (10)	H26B—C26'—H26C	109.5
H23A—C23—H23B	108.9 (11)	Si1—C25—H25A	109.5
C10—C15—H15	119.8	Si1—C25—H25B	109.5
C10—C15—C14	120.49 (11)	Si1—C25—H25C	109.5
C14—C15—H15	119.8	H25A—C25—H25B	109.5
C1—C2—H2	119.9	H25A—C25—H25C	109.5
C3—C2—C1	120.28 (11)	H25B—C25—H25C	109.5
C3—C2—H2	119.9	Si1—C26—H26D	109.5
C1—C6—H6	120.2	Si1—C26—H26E	109.5
C5—C6—C1	119.65 (11)	Si1—C26—H26F	109.5
C5—C6—H6	120.2	H26D—C26—H26E	109.5
C22—C17—S3	118.00 (10)	H26D—C26—H26F	109.5
C18—C17—S3	122.37 (10)	H26E—C26—H26F	109.5
C18—C17—C22	119.57 (12)	Si1—C24—H24A	109.5
C15—C10—S2	124.22 (9)	Si1—C24—H24B	109.5
C15—C10—C11	119.28 (11)	Si1—C24—H24C	109.5
C11—C10—S2	116.50 (10)	H24A—C24—H24B	109.5
C17—C22—H22	119.9	H24A—C24—H24C	109.5
C21—C22—C17	120.19 (13)	H24B—C24—H24C	109.5
C21—C22—H22	119.9	Si1'—C24'—H24D	109.5
C17—C18—H18	120.1	Si1'—C24'—H24E	109.5
C19—C18—C17	119.75 (13)	Si1'—C24'—H24F	109.5
C19—C18—H18	120.1	H24D—C24'—H24E	109.5
C15—C14—H14	119.9	H24D—C24'—H24F	109.5
C13—C14—C15	120.15 (13)	H24E—C24'—H24F	109.5
C13—C14—H14	119.9	Si1'—C25'—H25D	109.5
C2—C3—H3	119.8	Si1'—C25'—H25E	109.5
C4—C3—C2	120.38 (12)	Si1'—C25'—H25F	109.5
C4—C3—H3	119.8	H25D—C25'—H25E	109.5
C22—C21—H21	119.9	H25D—C25'—H25F	109.5
C20—C21—C22	120.13 (13)	H25E—C25'—H25F	109.5
C20—C21—H21	119.9	C23—O1'—Si1'	123.7 (7)
C6—C5—H5	119.6	C23—O1—Si1	123.6 (7)
			
S1—C1—C2—C3	−175.92 (10)	C23—C8—C7—S1	71.19 (9)
S1—C1—C6—C5	176.56 (10)	C23—C8—C9—S2	−169.94 (7)
S2—C10—C11—C12	179.49 (12)	C23—C8—C16—S3	−53.29 (11)
S3—C17—C22—C21	175.84 (9)	C15—C10—C11—C12	−0.3 (2)
S3—C17—C18—C19	−176.99 (9)	C15—C14—C13—C12	−0.2 (2)
C8—C23—O1'—Si1'	151.5 (9)	C2—C1—C6—C5	0.14 (18)
C8—C23—O1—Si1	163.9 (8)	C2—C3—C4—C5	0.1 (2)
C7—S1—C1—C2	−153.61 (8)	C6—C1—C2—C3	0.80 (18)
C7—S1—C1—C6	29.86 (11)	C6—C5—C4—C3	0.9 (2)
C7—C8—C9—S2	−49.35 (10)	C17—S3—C16—C8	−81.99 (9)
C7—C8—C16—S3	−172.59 (7)	C17—C22—C21—C20	1.09 (18)
C7—C8—C23—O1'	60.9 (7)	C17—C18—C19—C20	1.48 (19)
C7—C8—C23—O1	60.3 (8)	C10—S2—C9—C8	−157.51 (7)
C9—S2—C10—C15	−12.64 (11)	C10—C15—C14—C13	0.8 (2)
C9—S2—C10—C11	167.62 (10)	C10—C11—C12—C13	0.9 (2)
C9—C8—C7—S1	−47.38 (10)	C22—C17—C18—C19	0.02 (17)
C9—C8—C16—S3	65.28 (10)	C22—C21—C20—C19	0.4 (2)
C9—C8—C23—O1'	−177.2 (7)	C18—C17—C22—C21	−1.30 (17)
C9—C8—C23—O1	−177.8 (8)	C14—C15—C10—S2	179.72 (10)
C16—S3—C17—C22	129.90 (9)	C14—C15—C10—C11	−0.55 (18)
C16—S3—C17—C18	−53.05 (11)	C14—C13—C12—C11	−0.6 (3)
C16—C8—C7—S1	−169.08 (7)	C21—C20—C19—C18	−1.7 (2)
C16—C8—C9—S2	69.04 (9)	C26'—Si1'—O1'—C23	163.0 (10)
C16—C8—C23—O1'	−55.8 (7)	C25—Si1—O1—C23	47.9 (13)
C16—C8—C23—O1	−56.3 (8)	C26—Si1—O1—C23	167.5 (11)
C1—S1—C7—C8	−115.70 (7)	C24—Si1—O1—C23	−72.9 (12)
C1—C2—C3—C4	−0.9 (2)	C24'—Si1'—O1'—C23	−78.3 (11)
C1—C6—C5—C4	−1.0 (2)	C25'—Si1'—O1'—C23	43.2 (13)

### Selected geometric parameters for bromomethylalcohol 1 (Å, °).

**Table:**

Br1–C3	1.948 (3)	C1–C3–Br1	112.99 (18)
Br2–C4	1.952 (3)	C1–C4–Br2	113.48 (18)
Br3–C5	1.950 (3)	C1–C5–Br3	113.21 (19)
O1–C2	1.432 (3)	O1–C2–C1	111.6 (2)

### Selected geometric parameters for silylated thioether ligand 3 (Å, °).

**Table:**

S1–C7	1.8242 (11)	C1–S1–C7	105.84 (5)
S1–C1	1.7661 (11)	C10–S2–C16	103.67 (5)
S2–C9	1.8183 (11)	C17–S3–C16	104.74 (5)
S2–C10	1.7621 (12)	C23–O1–Si1	123.3 (8)
S3–C16	1.8211 (11)	C23–O1'–Si1'	123.8 (8)
S3–C17	1.7739 (13)		
O1–C23	1.385 (17)		
O1'–C23	1.480 (15)		
O1–Si1	1.636 (14)		
O1'–Si1'	1.669 (15)		
